# COVID-19 Vaccine Booster Shot Preserves T Cells Immune Response Based on Interferon-Gamma Release Assay in Inflammatory Bowel Disease (IBD) Patients on Anti-TNFα Treatment

**DOI:** 10.3390/vaccines11030591

**Published:** 2023-03-03

**Authors:** Grazia Pavia, Rocco Spagnuolo, Angela Quirino, Nadia Marascio, Aida Giancotti, Silvio Simeone, Cristina Cosco, Elena Tino, Federico Carrabetta, Gianfranco Di Gennaro, Carmelo Nobile, Aida Bianco, Giovanni Matera, Patrizia Doldo

**Affiliations:** 1 Unit of Clinical Microbiology, Department of Health Sciences, “Magna Græcia” University of Catanzaro—“Mater Domini” Teaching Hospital, 88100 Catanzaro, Italy; 2 Unit of Gastroenterology, Department of Clinical and Experimental Medicine, “Magna Græcia” University of Catanzaro—“Mater Domini” Teaching Hospital, 88100 Catanzaro, Italy; 3 Department of Health Sciences, School of Medicine, “Magna Græcia” University of Catanzaro, 88100 Catanzaro, Italy

**Keywords:** cellular immune response, humoral immune response, anti-SARS-CoV-2 immunogenicity, inflammatory bowel disease, anti-TNFα agents

## Abstract

Immune-modifying treatment in inflammatory bowel disease (IBD) impairs the humoral response. The role of T lymphocytes in this setting is still unclear. This study aims to assess if a booster shot (third dose) of BNT162b2 mRNA COVID-19 vaccine enhanced the humoral response and elicited cellular immunity in IBD patients on different immuno-therapy regimens compared to healthy controls (HCs). Five months after a booster dose, serological and T-cell responses were assessed. The measurements were described using geometric means with 95% confidence intervals. The differences between study groups were assessed by Mann–Whitney tests. Seventy-seven subjects (*n* = 53 IBD patients and *n* = 24 HCs), who were fully vaccinated and not previously SARS-CoV-2 infected, were recruited. Regarding the IBD patients, 19 were affected by Crohn’s disease and 34 by ulcerative colitis. During the vaccination cycle, half of the patients (53%) were on stable treatment with aminosalicylates, and 32% were on biological therapy. No differences in antibody concentrations between IBD patients and HCs, nor T-cell responses, were found. Stratifying IBD patients based on the type of treatment (anti-TNFα agents vs. other treatment regimens), a decrease only in antibody titer (*p* = 0.008), but not in cellular response, was observed. Even after the COVID-19 vaccine booster dose, the TNFα inhibitors selectively decreased the humoral immune response compared to patients on other treatment regimens. The T-cell response was preserved in all study groups. These findings highlight the importance of evaluating T-cell immune responses following COVID-19 vaccination in a routine diagnostic setting, particularly for immunocompromised cohorts.

## 1. Introduction

Inflammatory bowel disease (IBD), encompassing Crohn’s disease (CD) and ulcerative colitis (UC), are chronic progressive immune-mediated conditions that require lifelong medical treatment [1]. Aberrant immune responses resulting in disruption of the gastrointestinal mucosa, environmental factors, gut microbiota composition, and genetic predisposition contribute to disease evolution and to the health-related life quality of IBD patients [2,3,4,5,6,7]. New biological agents, such as anti-tumor necrosis factor-α (TNFα) (infliximab, adalimumab, golimumab), anti-integrin α_4_β_7_ (vedolizumab), anti-IL12/23 (ustekinumab) monoclonal antibodies and, most recently, small molecules (Janus kinase [JAK] inhibitors), have improved IBD management [8,9,10]. Although these regimens were the keystone of long-standing IBD disease remission by suppressing immune responses, they have raised concerns in the SARS-CoV-2 pandemic era [11,12]. Indeed, it is well known that anti-TNFα agents have variable effects on vaccine effectiveness, as already demonstrated for influenza, *pneumococcus*, tetanus, and viral hepatitis [13,14,15,16,17,18,19]. Such effects could not be found in IBD patients treated with vedolizumab or ustekinumab, which antagonize molecular mediators of inflammation distinct from the TNF pathway [20,21]. In the last two years, several studies have reported impaired humoral responses in IBD patients treated with infliximab or those treated with other immunosuppressants, compared to healthy controls (HCs) after one or two doses of approved COVID-19 vaccines (mainly mRNA vaccines or containing non-replicating viral vector) [22,23,24,25]. Furthermore, the serological response seems to decrease more rapidly in IBD patients on anti-TNFα inhibitors than those on vedolizumab [26,27,28]. Despite T lymphocytes being the key orchestrators of adaptive immune responses and conferring long-lasting protection through immune memory [29,30], their role in this setting is not well defined. In particular, the effects of immune-modifying treatments on cellular immune response in IBD patients following COVID-19 vaccination were poor and often controversial [31,32,33,34,35]. The imbalance of anti-SARS-CoV-2 vaccine effectiveness among two branches of adaptive immune responses could be due to the complexity of gold standard assays, which are unsuitable for clinical laboratory application. The introduction of validated assays for microbiology laboratories, such as the QuantiFERON SARS-CoV-2 Interferon-Gamma Release Assay (IGRA), could enable a more in-depth analysis of vaccine-elicited T-cells immunity, mainly for individuals that have a higher risk of developing severe COVID-19 [36,37,38,39].

The primary aim of our study was to evaluate if a third (booster) dose of BNT162b2 mRNA COVID-19 vaccine substantially enhanced the humoral response and elicited cellular immunity in IBD patients on different therapy regimens compared to healthy controls (HCs). The second objective was to explore the correlation between COVID-19 vaccine-elicited humoral and cellular immune responses in our study groups.

## 2. Materials and Methods

### 2.1. Study Design

Between May and August 2022, we performed an observational study on consecutive IBD outpatients attending the Unit of Gastroenterology at the “Mater Domini Hospital” in Catanzaro. A healthy population 2:1 matched pair case-control was also recruited. All enrolled subjects received primary series doses of the BNT162b2 mRNA COVID-19 vaccine (Pfizer-BioNTech, Mainz, Germany) plus a booster shot six months after the second dose. The IBD patients and HCs were over 18 years old, with no evidence of current or previous infection with SARS-CoV-2 detected by real-time PCR and anti-nucleocapsid (N)-positive antibody response. Demographic and anthropometric characteristics were obtained for all participants. The IBD patients were stratified into two groups: (i) IBD patients treated with anti-TNFα agents and (ii) IBD patients on other treatment regimens (aminosalicylates and vedolizumab). Patients treated with anti-integrin α_4_β_7_ monoclonal antibodies, such as vedolizumab, were included in the same group as aminosalicylates since there is no systemic modulating effect. Systemic steroids and azathioprine regimen groups were excluded from the statistical analysis due to insufficient representation. The humoral and cellular immune responses elicited by the COVID-19 vaccine were evaluated five months after the booster shot for both the IBD patient groups and HCs. The correlation between COVID-19 vaccine-elicited humoral and cellular immune responses in all three study groups previously considered was also evaluated.

### 2.2. IBD Patient Cohort

IBD patients had a confirmed diagnosis of UC or CD based on clinical, endoscopic, and histological criteria. All patients underwent a full evaluation of disease characteristics, including disease duration and disease activity evaluated by the Harvey–Bradshaw index (HBI) [40] for CD and the Mayo score (MS) for UC [41]. An HBI score > 7 and MS > 5 defined active chronic disease. Information about treatment regimens was also collected.

### 2.3. SARS-CoV-2 Humoral Immune Response

Quantitative evaluation of SARS-CoV-2 IgG anti-spike (S) glycoprotein antibodies was performed by the Liaison^®^ SARS-CoV-2 TrimericS IgG chemiluminescent immuno-assay (CLIA) on the Liaison XL (Diasorin^®^ S.P.A., Saluggia, Italy), according to the manufacturer’s instructions. The cut-off for positivity was 33.8 binding activity units (BAU)/mL. This assay showed an optimal correlation with the micro-neutralization test (negative and positive agreement of 100% and 96.9%, respectively) and was standardized against the WHO internal standard [42]. Regarding analytical performance, high sensitivity (98.7%) together with high specificity (99.5%) ensure accurate results. Samples containing levels of IgG anti-S antibodies above the measurement range (>2080 BAU/mL) were further diluted 1:10 using LIAISON^®^ TrimericS IgG Diluent. In order to exclude previous SARS-CoV-2 asymptomatic infection during the overall period considered, the anti-N response was determined using the Roche Elecsys^®^ Anti-SARS-CoV-2 electro-chemiluminescence immuno-assay (ECLIA) on the Cobas e 601 module (Roche^®^, Mannheim, Germany).

### 2.4. SARS-CoV-2 Cellular Immune Response

The specific cellular immune response was evaluated by the *in vitro* diagnostic test QuantiFERON^®^ SARS-CoV-2 (Qiagen^®^, Hilden, Germany). This IGRA assay is able to qualitatively evaluate the peripheral blood T lymphocyte response following stimulation with two SARS-CoV-2 S-derived peptide (Ag1 and Ag2) pools. The Ag1 and Ag2 blood collection tubes were coated on the inner walls with CD4^+^ epitopes derived from the S1 subunit (receptor binding domain—RBD), and CD4^+^ and CD8^+^ epitopes from the S1 and S2 subunits of the S protein, respectively. The IFNγ release was measured after 16–21 h of incubation at 37 °C by enzyme-linked immunosorbent assay (ELISA) on a personal lab system (Adaltis^®^, Rome, Italy). Background levels of INFɣ produced in the QuantiFERON^®^ SARS-CoV-2 Nil tube (negative control) without peptide stimulation were subtracted from INFɣ values of the Ag tubes. The immune competence of the subject cohort was addressed through a QuantiFERON^®^ SARS-CoV-2 mitogen tube (positive control). Cellular immune response was defined as an INFɣ value at least 0.15 IU/mL greater than the background value from the Nil tube.

### 2.5. Statistical Analysis

The IFNγ values after SARS-CoV-2 antigen pool stimulation (Ag1/Ag2) and IgG anti-S glycoprotein antibody concentrations were described using geometric means with 95% confidence intervals [95% CI]. The other continuous variables were described by mean and standard deviation when normally distributed, and by median and interquartile range when skewed. Categorical variables were expressed as counts and percentages. The normality distribution of continuous variables was verified with the Shapiro–Wilk test. The low sample size did not allow estimate adjustments by introducing other variables. Wilcoxon–Mann–Whitney tests were performed to investigate the significance of differences in humoral immunity (IgG anti-S glycoprotein antibodies titer) and cellular immune response (INFγ values induced by Ag1 and Ag2 peptide pools) between groups. Further, the Spearman rank coefficient (R) was calculated to investigate the correlation between two branches of adaptive immune response with the type of drug administered at the time of vaccination. Statistical analyses were performed by STATA.17.0 and GraphPad Prism 9.

## 3. Results

### 3.1. Cohort Characteristics

Demographic and clinical characteristics of the entire study population are shown in Table 1. Eighty-four subjects (*n* = 58 IBD patients and *n* = 26 HCs) were recruited in our study. Seven out of eighty-four individuals (9%) (*n* = 5 IBD patients and *n* = 2 HCs) with positive SARS-CoV-2 total Ig anti-N antibodies were excluded from the final analysis. No differences in age (52 years [42–65] vs. 50 years [39–63]), gender (males: *n* = 35, 66% vs. *n* = 14, 58%), or body mass index (BMI) (25 kg/m^2^ [22–28] vs. 24 [23–26]) were found among the two groups. Twenty-five percent (*n* = 13) and twenty-one percent (*n* = 5) of the IBD patients and HCs reported at least one comorbidity, respectively. Out of 53 IBD patients, 19 were affected by CD (36%), and 34 by UC (64%). Median IBD disease duration was 11 years [5–22], and most were in clinical remission with HBI < 7 for CD (4 [0–8]) and MS < 5 or UC (0 [0–0]). During the primary series doses of the COVID-19 vaccination, the majority of patients (*n* = 28, 53%) were on stable treatment with aminosalicylates, followed by 14 (27%) on anti-TNFα monoclonal antibodies (*n* = 5 infliximab, *n* = 6 golimumab, *n* = 3 adalimumab), while 5 (9%) were treated with anti-α_4_β_7_ integrin monoclonal antibodies (vedolizumab). Three (6%) patients were on systemic steroids as well as azathioprine treatment. At five months after the booster dose, no changes in the treatment regimen had been made in any of the IBD patients.

### 3.2. SARS-CoV-2 Vaccine-Induced Immune Response in IBD Patients vs. HCs

**Figure 1:**
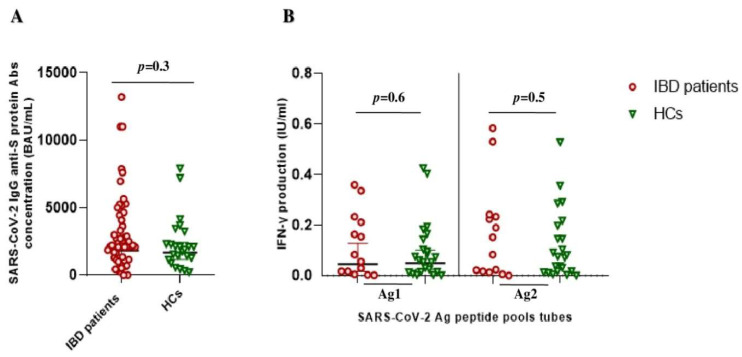
SARS-CoV-2 vaccine-elicited adaptive immune response after booster shot in IBD patients and healthy controls (HCs). (**A**) Anti-SARS-CoV-2 trimeric S protein antibodies (Abs) concentration (BAU/mL) between the IBD patients (red circles) and HCs (green triangles) groups. (**B**) QuantiFERON SARS-CoV-2 antigen tubes (Ag1 and Ag2) response, express as IFN-γ production (IU/mL), among the IBD patients (red circle) and HCs (green triangle) groups.

Five months after the vaccine booster dose, all subjects (except one) showed a good humoral response (geometric mean 2105 BAU/mL [95% CI 1215–3532]). No significant difference in the geometric mean of anti-SARS-CoV-2 IgG titers between IBD patients and HCs was found (1829 BAU/mL [1226–2729] vs. 1676 BAU/mL [1156–2429]; *p* = 0.3) (Figure 1A). The same trend was observed for the cellular immune response, with overlapping geometric means of IFNγ production in both Ag1 (0.06 IU/mL [0.03–0.1] vs. 0.04 IU/mL [0.02–0.1]; *p* = 0.6) and Ag2 (0.09 IU/mL [0.05–0.14] vs. 0.07 IU/mL [0.03–0.14]; *p* = 0.5) tubes, respectively (Figure 1B).

**Figure 2:**
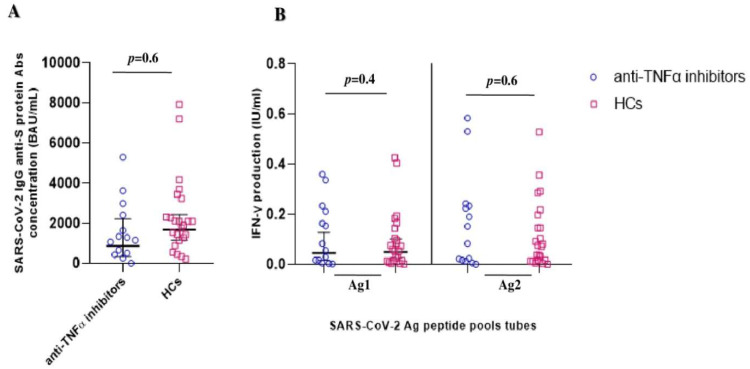
SARS-CoV-2 vaccine-elicited adaptive immune response after booster shot in IBD patients on anti-TNFα treatments and healthy controls (HCs). (**A**) Anti-SARS-CoV-2 trimeric S protein antibody concentration (BAU/mL) response between the IBD patients on anti-TNFα treatments (blue circles) and HCs (violet squares) groups. (**B**) QuantiFERON SARS-CoV-2 antigen tubes (Ag1 and Ag2) response, expressed as IFNγ production (IU/mL), among the IBD patients (blue circles) and HCs (violet squares) groups.

Furthermore, no significant differences were observed when comparing HCs with IBD patients only treated with anti-TNFα agents, both in humoral immune response (1257 BAU/mL [744–2124] vs. 1676 BAU/mL [1156–2429]; *p* = 0.6] and in the QuantiFERON SARS-CoV-2 Ag1 (0.06 IU/mL [0.02–0.19] vs. 0.04 IU/mL [0.02–0.1]; *p* = 0.4) and Ag2 (0.09 IU/mL [0.03–0.26] vs. 0.07 IU/mL [0.03–0.14]; *p* = 0.6) responses, respectively (Figure 2).

### 3.3. SARS-CoV-2 Vaccine-Induced Immune Response in IBD Patients on TNFα Inhibitors vs. Others Treatment Regimens

**Figure 3:**
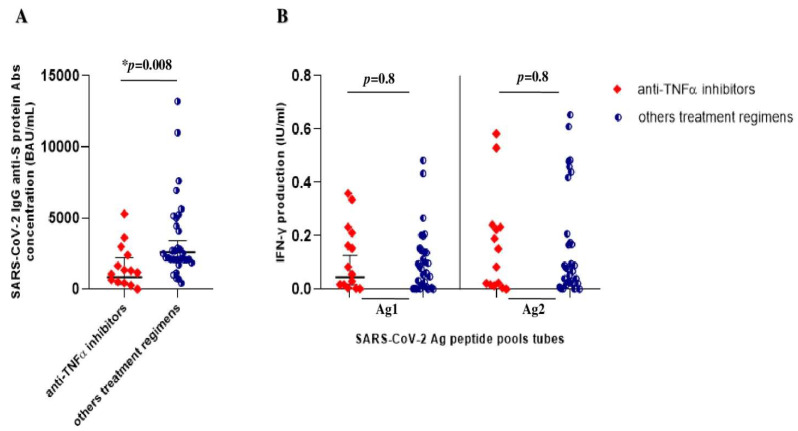
SARS-CoV-2 vaccine-elicited immune response after booster shot in IBD patients onanti-TNFα treatment and other treatment regimens. (**A**) Anti-SARS-CoV-2 trimeric S protein antibody concentration (BAU/mL) in IBD patients on anti-TNFα treatments (red rhombs) vs. other treatment regimens (aminosalicylates and vedolizumab) (blue circles). (**B**) QuantiFERON SARS-CoV-2 antigen tube (Ag1 and Ag2) responses, express as IFN-γ production (IU/mL), in IBD patients on anti-TNFα treatments (red rhombs) vs. other regimens (aminosalicylates and vedolizumab) (blue circles). Legend: the * highlight the significance of *p*-value < 0.05.

As shown in Figure 3, we further compared immunogenicity in IBD patients on anti-TNFα agents vs. those undergoing other treatments. The geometric means of IgG anti-S glycoprotein antibody concentrations in IBD patients on anti-TNFα agents were lower than those in patients on other treatment regimens, 858.6 BAU/mL [332.6–2216.5] vs. 2612.7 BAU/mL [1999.6–3413.7] (*p* = 0.008), respectively (Figure 3A).

Regarding the cellular immune response, we observed overlapping values of IFNγ production after SARS-CoV-2 antigen stimulation of whole blood in both groups. The IBD patients treated with anti-TNFα agents showed an IFNγ geometric mean value after SARS-CoV-2 Ag1 stimulation of 0.11 IU/mL [0.04–0.18], compared to 0.13 IU/mL [0.06–0.2] the non-biological regimen group. The same values were observed following SARS-CoV-2 Ag2 stimulation; the anti-TNFα group showed an IFNγ geometric mean of 0.15 IU/mL [0.05–0.2], equivalent to other treatment regimens (0.19 IU/mL [0.1–0.3]. No statistical significance was observed in T-cell immune response comparing both groups (*p* = 0.8) (Figure 3B).

### 3.4. Correlation between COVID-19 Vaccine-Elicited Humoral and Cellular Immune Responses

The SARS-CoV-2 IgG anti-S antibody titers and QuantiFERON SARS-CoV-2 antigen responses significantly correlated with the healthy control group and IBD patients treated with aminosalicylates and vedolizumab for both SARS-CoV-2 antigens. No significant correlation between humoral and cellular immune responses was observed in the IBD patients on anti-TNFα agents for both SARS-CoV-2 antigens (Table 2).

## 4. Discussion

How immuno-suppressive agents in IBD can impair adaptive immune responses to SARS-CoV-2 vaccination is pivotal for the management of severe COVID-19 outcomes due to highly transmissible and immune-escape viral variants. This observational study provides findings on COVID-19 booster-elicited humoral and cellular immune responses in a cohort of IBD patients on different immune-modifying treatments compared to HCs with no previous SARS-CoV-2 infection. Hence, we showed that IBD patients on each treatment regimen obtained a good serological response, with no significant differences compared to HCs, following a COVID-19 vaccine booster shot. This result supports the decision, currently being offered by some countries, of implementing booster doses in different immunocompromised cohorts to enhance immunogenicity. However, IBD patients on anti-TNFα inhibitors showed lower serological responses when compared to patients on other treatment regimens, even after five months following the booster dose. No influence on the adaptive immune response to the vaccine was exerted by aminosalicylates and vedolizumab. These findings are in line with observations after one or two doses of mRNA or non-replicating viral vector anti-SARS-CoV-2 vaccines [21,22,23,24]. Indeed, in IBD, vaccine effectiveness is strictly impaired by therapy with anti-TNFα inhibitors and small molecules (such as JAK inhibitors) [21,22,23,24]. More recently, Alexander and colleagues in the VIP cohort reported a boost in antibody response in IBD patients after the third dose of homologous (three doses of an mRNA vaccine) or heterologous vaccine schedules (e.g., two doses of adenovirus vaccine followed by one dose of mRNA vaccine). However, this response persisted significantly impaired in IBD patients on infliximab and tofacitinib, a second-generation selective JAK inhibitor targeting the JAK1 enzyme [35]. Adaptive immunity response is due not only to the action of specific antibodies but mainly to synergic responses of CD4^+^ and CD8^+^ T-cells, which by secreting immunostimulant cytokines, including IFNγ, play a key function in eliminating virus-infected cells [29]. Their role in this cohort is not yet well defined. No significant differences in T-cell response, following stimulation by both QuantiFERON Ag1 and Ag2 peptide pool tubes, between IBD patients and HCs were observed. The same results were found when stratifying patients by treatment (anti-TNFα vs. other treatment regimens). Previously, in line with our observations, Reuken and colleagues reported that after the first and second doses of mRNA or non-replicating viral vector vaccines, the T-cell response of a small IBD patient cohort was comparable to those seen in HCs with no influence due to treatment [31]. Similar insights were highlighted in a large previous study, where a good cellular immune response and no differences after the COVID-19 vaccine in IBD patients treated with infliximab vs. those treated with vedolizumab [31] were shown. Opposite findings were reported by Li D. et al., who evaluated 303 subjects fully immunized with mRNA and non-replicating viral vector vaccines, quantifying the breadth and depth of the T-cell clonal response at 8 weeks following two dose administration [34], showing that immune-modifying therapies selectively influenced the T-cell responses. Notably, the T-cell response was conserved in IBD patients treated with biological treatments targeting IL-12/23 and integrins and, contrary to expectations, was increased in cases given anti-TNFα therapy [34]. Recently, in agreement with our findings, the VIP study cohort reported that T-cell responses were not impaired compared to HCs. Nevertheless, a decreasing cellular immune response was observed in IBD patients treated with tofacitinib only. No effect on T-cell responses in patients on anti-TNFα agents was reported [35]. Unlike our study, most scientific reports evaluated humoral and cellular immune responses at slightly earlier time points post-vaccination than we did, when responses are likely to be at their highest. Indeed, it has been demonstrated that the serological response decreases more rapidly in IBD patients on anti-TNFα inhibitors [25,26,27], but the kinetics of the T-cell response is currently unknown. In our study we evaluated vaccine-elicited immunogenicity at five months following the booster shot. Our results for cellular immune response in IBD patients on different immune-modifying treatments at five months following the third vaccine dose might reflect more long-lasting protection by the T-cell immune memory. Furthermore, we showed an interesting finding, by Spearman rank correlation, which highlighted a significant positive correlation between humoral and cellular immune responses in HCs and in IBD patients treated with aminosalicylates and vedolizumab but not in IBD patients treated with anti-TNFα inhibitors (Table 2). These insights might suggest that, even after a booster dose, the interplay between humoral and cellular immunogenicity, in this cohort, might be impaired.

Since cellular immunity is not routinely determined, these results could help to understand the correlation between humoral and cellular immune responses and could contribute to better management of these immunocompromised patients on anti-TNFα inhibitors in the COVID-19 setting. In this regard, the application of an automated and validated test, such as the QuantiFERON SARS-CoV-2 IGRA assay, to evaluate T cell responses in a large number of clinical samples, allowed us to produce results within 24 h with a good correlation to gold standard tests (ELISpot assays), as already has been demonstrated [35,36] and to interpret results objectively based on a predefined cut-off.

Some limitations of this study should be reported. First, adaptive immune responses were measured at a single time point, and we are not able to comment on the kinetics of serological and cellular immunity over time following booster doses of the COVID-19 vaccine. Second, although we have accounted for the need to evaluate, in a clinical setting, the cellular immune response in a large number of samples within 24 h, we understand that our study does not allow assessment of repertoire diversity and clonal size, important factors in protective T-cell immunity. Third, the small size of our study cohort might fail to detect a significant difference in adaptive immunogenicity in the different patient subgroups and did not allow us to adjust the estimates for confounders (e.g., smoking and comorbidities). Indeed, we were not able to stratify patients into subgroups according to different types of treatments. Hence, we stratified IBD patients into only two categories: (i) IBD patients treated with anti-TNFα agents and (ii) IBD patients on other treatment regimens (aminosalicylates and vedolizumab). Since there is no systemic modulating effect for patients treated with vedolizumab, they were included in the same group as aminosalicylates. Treatments with systemic steroids and azathioprine regimen groups were excluded from our statistical analysis due to insufficient numbers. In addition, in our IBD outpatients attending the Unit of Gastroenterology from “Mater Domini Hospital” in Catanzaro, we did not include patients on JAK inhibitors or ustekinumab. The booster effect of the vaccine on patients treated with these agents was not examined.

## 5. Conclusions

In a routine clinical setting, our findings could be pivotal in evaluating both humoral and T-cell immune responses after the COVID-19 vaccine, particularly for patients treated with immune-modifying medications. These insights could provide rapid indications of the vaccine-elicited immunogenicity of immunocompromised patients and their correlation with protective immunity. Moreover, in view of future COVID-19 vaccines able to elicit T-cell immunity against SARS-CoV-2, these results could be useful in the appropriate stratification of high-risk populations.

## Figures and Tables

**Table 1:** Demographic and clinical characteristics of the study cohort.

Characteristics	IBD Patients (*n* = 53)	HCs (*n* = 24)
**Gender, male (*n*, %)**	35 (66)	14 (58)
**Age (median [IQR], years)**	52 [42–65]	50 [39–63]
**BMI (median [IQR], kg/m^2^)**	25 [22–28]	24 [23–26]
**Active smokers (*n*, %)**	5 (9)	5 (21)
**Type of disease (*n*, %)**		
Crohn’sdisease	19 (36)	
Ulcerative colitis	34 (64)	
**Disease activity (median [IQR])**		
HBI score	4 [0–8]	
MS score	0 [0–0]	
**Disease localization (*n*, %)**		
*Crohn’s disease*		
L1 (ileal)	1 (47)	
L2 (colonic)	4 (21)	
L3 (ileocolonic)	6 (32)	
*Ulcerative colitis*		
E1 (proctitis)	7 (21)	
E2 (left-sided)	4 (12)	
E3 (extensive)	23 (68)	
**Disease duration (median [IQR], years)**	11 [5–22]	
**Treatment regimens (*n*, %)**		
aminosalicylates *	28 (53)	
**systemic steroids**	3 (6)	
**azathioprine**	3 (6)	
*anti-TNFα*	14 (27)	
infliximab	5 (10)	
adalimumab	3 (6)	
golimumab	6 (11)	
*vedolizumab*	5 (9)	
**Comorbidities (*n*,%)**		
None	32 (60)	18 (75)
1	13 (25)	5 (21)
2	7 (13)	1 (4)
3	1 (2)	0 (0)

* Some patients were receiving aminosalicylates in association with other treatments; IBD = inflammatory bowel diseases; BMI = body mass index; HBI score = Harvey–Bradshaw index for CD severity (0–16); MS score = Mayo index for UC severity (0–12) anti-TNFα = monoclonal antibody anti-TNFα; comorbidities = cardiological diseases, neurological disorders, diabetes.

**Table 2:** Spearman rank correlation coefficient (R) and significance (*p*-value) between humoral and cellular immune response for Ag1 and Ag2 peptide pool. Significant correlations (*p*-value < 0.05) are indicated in bold.

Study Cohort Groups	Spearman’s Rank (R, *p*-Value)	
	*IgG*/*Ag1*	*IgG*/*Ag2*
**Healthy controls**	**0.5**	**0.6**
	(*p* = 0.009)	(*p* = 0.001)
**IBD on anti-TNF** **α** **agents**	0.3	0.3
	(*p* = 0.2)	(*p* = 0.4)
**IBD on vedolizumab**	**0.4**	**0.4**
	(*p* = 0.01)	(*p* = 0.04)

## Data Availability

The raw data supporting the conclusions of this article will be made available by the authors, without undue reservation.
